# A comprehensive aligned *nifH* gene database: a multipurpose tool for studies of nitrogen-fixing bacteria

**DOI:** 10.1093/database/bau001

**Published:** 2014-02-05

**Authors:** John Christian Gaby, Daniel H. Buckley

**Affiliations:** Department of Crop and Soil Sciences, Cornell University, Ithaca, NY 14853, USA

## Abstract

We describe a nitrogenase gene sequence database that facilitates analysis of the evolution and ecology of nitrogen-fixing organisms. The database contains 32 954 aligned nitrogenase *nifH* sequences linked to phylogenetic trees and associated sequence metadata. The database includes 185 linked multigene entries including full-length *nifH*, *nifD*, *nifK* and 16S ribosomal RNA (rRNA) gene sequences. Evolutionary analyses enabled by the multigene entries support an ancient horizontal transfer of nitrogenase genes between *Archaea* and *Bacteria* and provide evidence that *nifH* has a different history of horizontal gene transfer from the *nifDK* enzyme core. Further analyses show that lineages in nitrogenase cluster I and cluster III have different rates of substitution within *nifD*, suggesting that *nifD* is under different selection pressure in these two lineages. Finally, we find that that the genetic divergence of *nifH* and 16S rRNA genes does not correlate well at sequence dissimilarity values used commonly to define microbial species, as stains having <3% sequence dissimilarity in their 16S rRNA genes can have up to 23% dissimilarity in *nifH*. The *nifH* database has a number of uses including phylogenetic and evolutionary analyses, the design and assessment of primers/probes and the evaluation of nitrogenase sequence diversity.

**Database URL**: http://www.css.cornell.edu/faculty/buckley/nifh.htm

## Introduction

Biological nitrogen fixation contributes around half of annual nitrogen inputs into the biosphere (1) and is an important source of nitrogen in natural ecosystems (2) and also in agricultural systems (3, 4). Biological nitrogen fixation is catalyzed by nitrogenase, a complex enzyme that has two components, a heterotetrameric core encoded by *nifD* and *nifK*, and a dinitrogenase reductase subunit encoded by *nifH* (5). Dinitrogenase reductase transfers reducing equivalents to the core enzyme complex where those reducing equivalents are used to convert N_2_ into NH_3_ equivalents. The *nifH* gene is the biomarker most widely used to study the ecology and evolution of nitrogen-fixing bacteria (6). Surveys of *nifH* diversity have been conducted in a wide range of environments including various marine (7–9), terrestrial (10–12) and hydrothermal sites (13) among others. The diversity of nitrogen-fixing organisms varies dramatically across habitats with different habitats selecting for different groups of nitrogen-fixing organisms (14, 15).

Homologs of the *nifH* gene can be divided into five main phylogenetic clusters (16). Cluster I contains a diverse group of *nifH* genes primarily from aerobic and facultatively anaerobic organisms that belong to phyla including *Proteobacteria*, *Cyanobacteria*, *Firmicutes* and *Actinobacteria*. Cluster III contains *nifH* genes that are almost exclusively found in obligate anaerobes including methanogenic *Archaea*, *Treponema*, *Clostridium* and sulfate-reducing and sulfur-reducing species of *Deltaproteobacteria*. Cluster II contains *anfH*, alternative nitrogenases that are paralogs of *nifH* and use an Fe–Fe cofactor in place of the Fe–Mo cofactor used by *nifH* (17). There also exist V–Fe alternative nitrogenases encoded by *vnfH*. The alternative nitrogenases appear to be found only in the genomes of organisms that also contain *nif* genes (17). Clusters IV and V contain paralogous genes that do not participate in nitrogen fixation (6, 18–21).

Analyses of microbial gene sequences that serve as functional biomarkers are facilitated by the availability of annotated databases of aligned sequences. Aligned sequences are required for phylogenetic analyses [e.g. (6, 22–24)], diversity analyses [e.g. (15)] and the design and evaluation of polymerase chain reaction (PCR) primers and probes [e.g. (25, 26)]. Universal and group-specific PCR primers are used in quantitative real-time PCR for the quantification of gene copy numbers in the environment [e.g. (27–29)], for expression studies (30) or for tracking organisms [e.g. (27, 31)]. Hence, many contemporary techniques in molecular microbial ecology require PCR and specific primers, which must be designed and evaluated using annotated sequence databases.

There is strong demand for the creation of a centralized, well-described, aligned and vetted *nifH* database. Similar databases of 16S ribosomal RNA (rRNA) gene sequences have been of great utility in the field of microbial ecology (32–34). In this work, we describe a comprehensive *nifH* database, a multipurpose tool to facilitate the work of researchers who use sequence-based techniques to study nitrogen-fixing bacteria. The database is implemented in the ARB software package (35) that allows for the compilation and association of aligned sequence data, sequence metadata and phylogenetic trees. Phylogenetic trees can be used to navigate the sequences and to explore phylogenetic patterns found in associated metadata. Preliminary versions of this *nifH* database, one containing 16 989 and another 23 843 sequences, were used to evaluate the diversity of *nifH* genes in different environments (15) and to evaluate PCR primers used in environmental surveys of *nifH* diversity (25). Here we release an updated *nifH* database containing 32 954 aligned *nifH* sequences and homologs along with guide trees. The new release also incorporates *nifD*, *nifK* and 16S rRNA genes from 185 sequenced genomes. These new data layers facilitate improved evolutionary analysis and enable assessment of ancestral patterns of horizontal gene exchange. In addition, the availability of full-length multigene sequence entries in the database, organized in a phylogenetic framework, will be of utility for the analysis of nitrogenase gene sequences recovered from metagenomic studies. The database and its associated description will be useful for studying the evolution, phylogeny and diversity of nitrogen-fixing bacteria.

### Database construction and analysis

The database comprises all *nifH* sequences available in the Genbank nucleotide database as of 16 May 2012. The majority of sequences are derived from environmental surveys of *nifH*, although the database also contains sequences obtained from a range of isolates. Sequence alignment was performed using the ARB integrated aligner used through an iterative process to generate a nucleotide alignment consistent with the Pfam (36) Fer4_NifH amino acid alignment. The Pfam Fer4_NifH (PF00142) alignment was used as an alignment template, reverse translated and imported into the ARB environment. This alignment template was used to generate a seed alignment consisting of a wide phylogenetic diversity of full-length and near full-length *nifH* nucleotide sequences from Genbank. The reverse-translated sequences were then deleted from the database, the seed alignment was manually vetted and then this seed alignment was used to complete the alignment of all remaining sequences with the ARB-integrated aligner. The final alignment was vetted manually, and low-quality sequences were removed.

Multigene database entries for *nifH*, *nifD*, *nifK* and 16S rRNA genes were generated using data from sequenced genomes. Aligned nucleic acid sequences were imported into ARB and merged as separate alignments linked to their corresponding *nifH* records. For import of the 16S rRNA gene sequences, we used the Greengenes (34) alignment, which allowed us to import the 16S rRNA helix data from the Greengenes ARB database. Alignments for NifD and NifK were generated using Clustal (37). The database may be downloaded in ARB database format at (http://www.css.cornell.edu/faculty/buckley/nifH_database_2012.arb), and a flat text version of the database that contains only aligned *nifH* sequences is also available (http://www.css.cornell.edu/faculty/buckley/nifH_database_2012.fasta).

Analyses of sequence characteristics and metadata were performed in ARB. Sequence lengths and G + C content were calculated using the ARB Node Display Setup. The start and end positions of sequences were calculated using the *nifH* gene of *Azotobacter vinelandii* (Genbank ACCN number M20568) as the reference sequence to provide sequence position. Sequence metadata was analyzed using the search and query function of ARB. Environmental categories were defined by the isolation source for specific sequences. Sequences belonging to the individual environmental categories are saved as configurations in the ARB *nifH* database and can be evaluated with respect to sequence phylogeny. There were 4759 sequences that lacked metadata relating to isolation source.

Evolutionary distances (E_d_) were calculated in ARB without distance correction or weighting. A column filter was developed to mask nucleotides for which position homology could not be determined, and this filter is available within the *nifH* database. Phylogenetic trees were constructed by neighbor-joining analysis of nucleotide alignments using Jukes–Cantor distance correction and a column filter to remove positions of uncertain homology. All trees are available in the *nifH* database. The database contains a large phylogenetic tree for the purpose of database navigation and sequence selection. This guide tree contains nonoverlapping sequences, and it was generated by first constructing a neighbor-joining tree using all available overlapping sequences and then inserting partial sequences using the quick add by parsimony feature of ARB. All statistical analyses were performed using The *R* Project for Statistical Computing (38).

### Analysis of database content

The database consists of 32 954 aligned sequences, the majority of which are *nifH*, although we have included some paralogous sequences for reference purposes. The *nifH* gene is one of the most heavily sequenced functional genes (Supplementary Figure S1) and >90% of *nifH* sequences in the database have been deposited since 2005 (Supplementary Figure S2). There are 375 full-length *nifH* genes in the database including 245 from sequenced genomes*.* The full-length *nifH* sequences represent 294 different strains and 222 different species. The majority of sequences have been determined from amplified fragments of the *nifH* gene, although the database also contains shotgun metagenome reads deposited in the Genbank nucleotide database.

The main phylogenetic tree associated with the database contains 32 931 sequences. There are 7030 sequences (21.35% of the total database) in *nifH* cluster III and subcluster IA (Supplementary Figure S6), the clusters that contain primarily obligate anaerobes. There are 24 799 sequences (75.31%) in *nifH* cluster I, which contains primarily aerobes and facultative anaerobes. The database also contains 578 sequences (1.76%) in *nifH* cluster II that comprises the iron-dependent alternative nitrogenase cluster, and 524 sequences in cluster IV that contains genes not functionally associated with nitrogenase.

Sequences in the database are derived from 1211 studies (Supplementary Figure S3), each contributing between 1 and 1299 sequences to the database. There are 584 studies that contributed only 1 sequence (48% of the total number of studies and 1.8% of the sequences in the database), and these represent sequences that originate from isolated strains. There are two studies contributing 1299 and 1297 sequences that originate from an analysis of nitrogen-fixing organisms associated with maize (10), while a study with 1257 sequences focused on coastal microbial mats (39). The 10 studies that contributed the most sequences account for 8516 sequences in total (26% of all sequences in the database), and the top 35 studies contributed 50% of all sequences.

**Figure 1.**
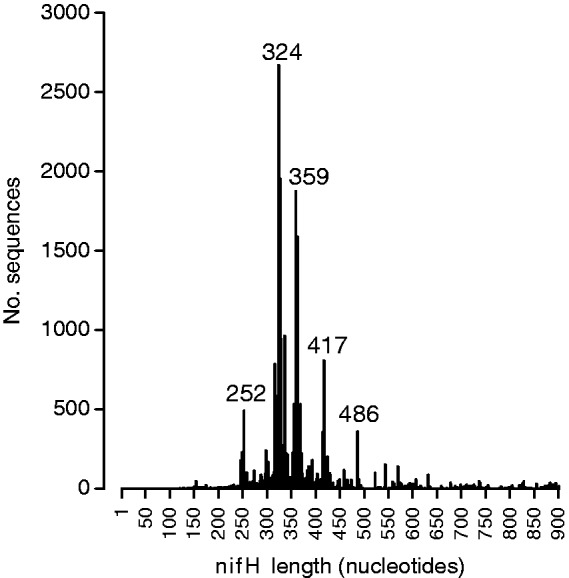
Number of *nifH* sequences in the database as a function of their length. The values of sequence length that are most frequent are labeled in the figure.

**Figure 2.**
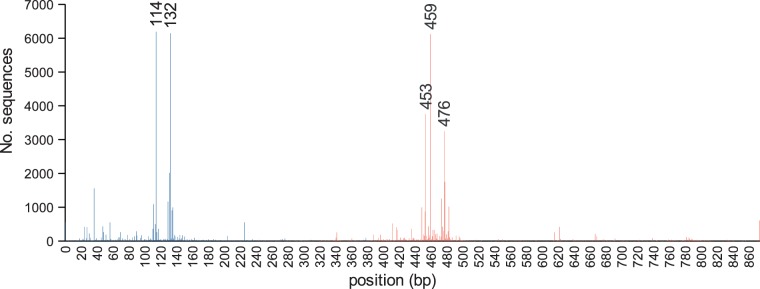
Count of start (blue) and stop (red) positions for the *nifH* sequences in the database. Nucleotide positions are numbered relative to the *A. vinelandii nifH* gene (Genbank ACCN number M20568).

These diverse studies have used a range of PCR primers to amplify *nifH* from environmental samples, and as a consequence, the *nifH* sequences in the database have a wide range of lengths. The mode fragment length for *nifH* gene sequences in the database is 324 bases (Figure 1). We have identified the frequency of different sequence start and end positions within the database. The most common start position for *nifH* primers is at ∼114 bases from the start codon, and the most common stop position at ∼459 bases from the start codon (Figure 2). The distance between the two most abundant start positions as well as stop positions (Figure 2) is 18 and 17 bases, which corresponds to the length of a primer, suggesting that in many cases primer sequences have not been trimmed from deposited sequences.

The *nifH* sequence representation varies greatly with respect to environmental origin (Supplementary Table S1). The environments represented by the most sequences are terrestrial (17 340) and marine (5958). Sequences from agricultural systems (7308) are represented heavily in the database. A total of 3082 *nifH* sequences have been reported from soils used for cultivation of maize, whereas 854 have been reported from soils used for cultivation of rice. Other terrestrial environments that have been sampled for *nifH* sequences include forests (2534 sequences) and grasslands (416 sequences). In addition, 978 *nifH* sequences are reported from freshwater environments such as lakes and rivers. Major anoxic environments from which *nifH* sequences have been sampled include microbial mats, flooded rice paddy soils, wetlands, sediments and termite guts. There are 19 445 sequence records (59% of the 32 954 total sequences) that document country of origin. It is clear that current sampling efforts underrepresent Africa and Oceana relative to other regions (Supplementary Figure S4A). Two studies of Brazilian maize (12) account for 79% of the sequences from South America. Of the top 10 countries for which sequences are available, the Netherlands (3502) and Brazil (3261) are most highly represented (Supplementary Figure S4B).

The mean G + C content for all *nifH* gene sequences in the database is 56.8%; however, the G + C content distribution of the *nifH* gene fragments is bimodal (Supplementary Figure S5). The number of sequences in the lower part of the distribution (G + C content of ≤53%) is 9543 and represents 29% of the *nifH* sequences in the database (Supplementary Figure S6). The low G + C content sequences primarily comprise *Cyanobacteria* (5157 sequences) and *Firmicutes* (887 sequences). Both of these phylogenetic groups are well known to contain organisms with low genome G + C content. The low G + C sequences also include some *Proteobacteria* (1361 sequences), cluster II sequences (330 sequences) and cluster IV sequences (254 sequences).

### Analysis of multigene entries

#### Comparative analyses of nifH, nifD and nifK

**Figure 3.**
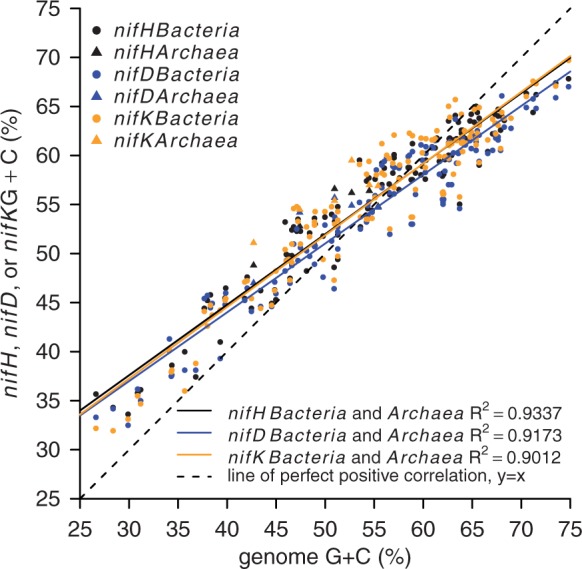
Sequence G + C content for complete genomes and the G + C content of corresponding *nifH*, *nifD* and *nifK* genes for 6 *Archaea* and 131 *Bacteria*. Colors represent different genes, and symbols indicate *Bacteria* and *Archaea* as shown in the figure legend.

The database contains 185 multigene entries composed of *nifH*, *nifD*, *nifK* and 16S rRNA genes. These multigene database entries were used to analyze the G + C content and E_d_ for *nif* genes. A linear relationship was observed between genome G + C content and *nif* gene G + C content (Figure 3; the *R*^2^ is 0.93, 0.92 and 0.90 for *nifH*, *nifD* and *nifK*, respectively), and outliers indicative of recent horizontal gene transfer were not observed (Figure 3).

**Figure 4.**
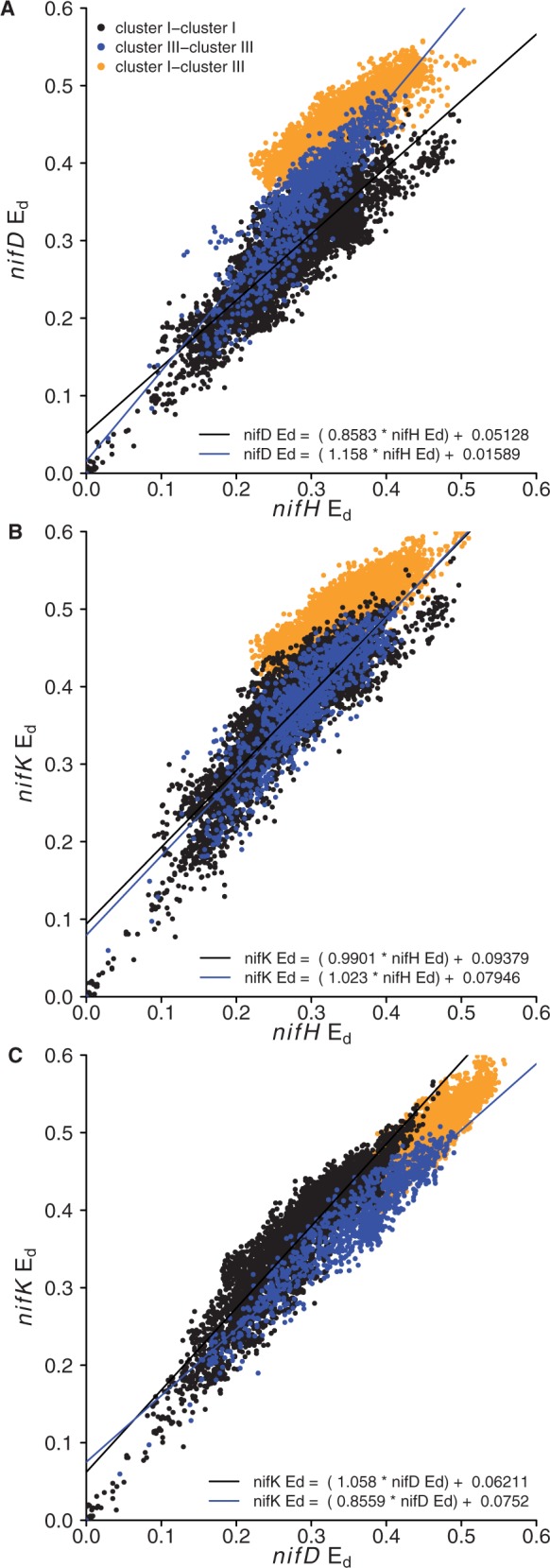
Comparison of relative E_d_ for *nifH* and *nifD* (**A**), *nifH* and *nifK* (**B**), *nifD* and *nifK* (**C**). The equation for the regression lines are labeled in each panel. Intra-cluster and inter-cluster comparisons are indicated with different colors as defined in the legend.

E_d_ values for the *nifHDK* genes from the 185 genomes were determined (Figure 4). Divergent patterns of E_d_ provide evidence for horizontal transfer and mutation rate variation between genes (40). The inter-cluster (cluster I relative to cluster III) divergence of *nifH* was less than expected relative to the pattern of intracluster divergence for either *nifD* (Figure 4A) or *nifK* (Figure 4B). In contrast, inter-cluster divergence of *nifD* and *nifK* corresponded well with intra-cluster patterns of divergence (Figure 4C). These data suggest the presence of an ancient horizontal gene transfer of *nifH* between cluster I and cluster III diazotrophs (Figure 4A and B), which did not involve transfer of *nifDK*.

The data also reveal *nifD* substitution rate variation between cluster I and cluster III diazotrophs (Figure 4). The rate variation is revealed on E_d_ plots by differences in the intracluster divergence of *nifD* relative to *nifH* and *nifK* (Figure 4A and C). The slope for the divergence of *nifD* relative to *nifH* is lower in cluster I than in cluster III, and this difference is significant (*f* = 294.3, *P* < 2 × 10^−^^16^, Figure 4A). Likewise, the slope for the divergence of *nifD* relative to *nifK* is lower in cluster I than in cluster III and this difference is also significant (*f* = 328.4, *P* < 2 × 10^−^^16^, Figure 4C). In contrast, there is no difference between cluster I and cluster III diazotrophs with respect to the slope of divergence for *nifH* and *nifK* (*f* = 3.451, *P* = 0.0633; Figure 4B). These results indicate that *nifD* is accumulating substitutions more slowly in cluster I diazotrophs than in cluster III diazotrophs.

#### Comparative analysis of nif genes and 16S rRNA genes

**Figure 5.**
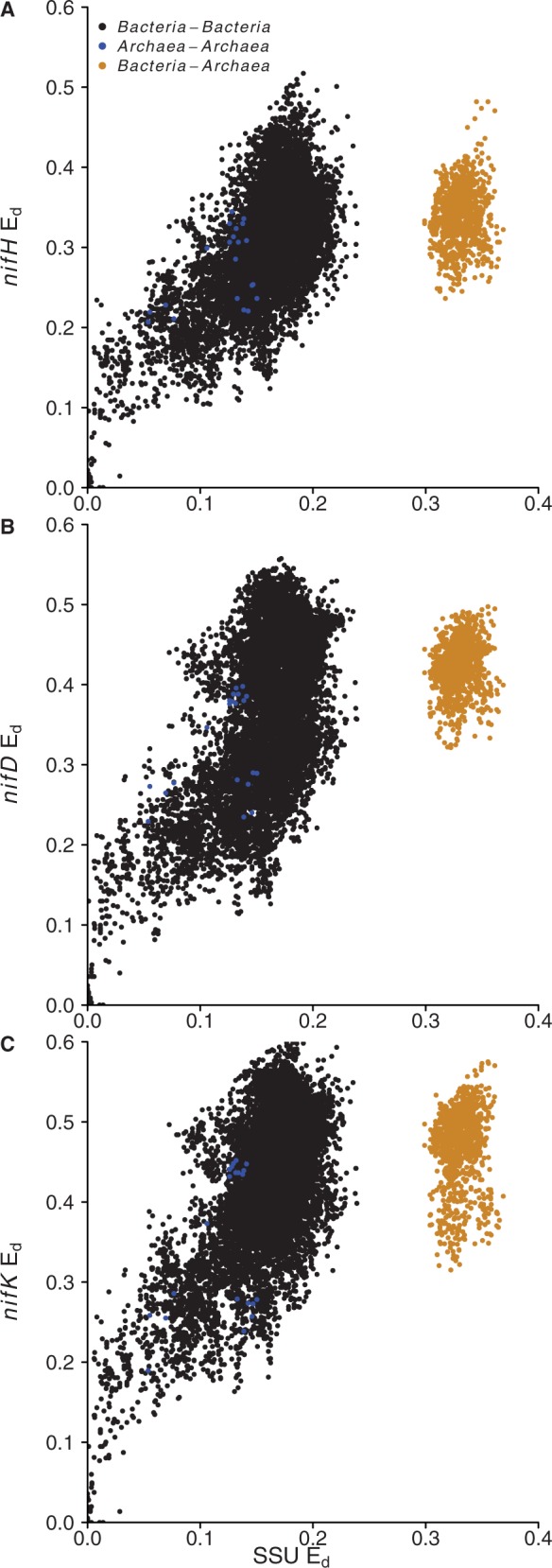
Comparison of relative E_d_ between 16S rRNA genes and *nifH* (**A**), *nifD* (**B**) and *nifK* (**C**). Colors indicate intra-domain and inter-domain comparisons for each set of genes as indicated in the figure legend.

E_d_ were calculated by pairwise comparison of 16S rRNA and *nif* genes from the 185 genomes (Figure 5). There is clear evidence for horizontal gene transfer of *nif* genes between *Bacteria* and *Archaea* (Figure 5). *Archaea*–*Bacteria* comparisons clearly partition from comparisons made within domains, and this can only occur as the result of *nif* genes horizontal transfer between *Bacteria* and *Archaea*.

**Figure 6.**
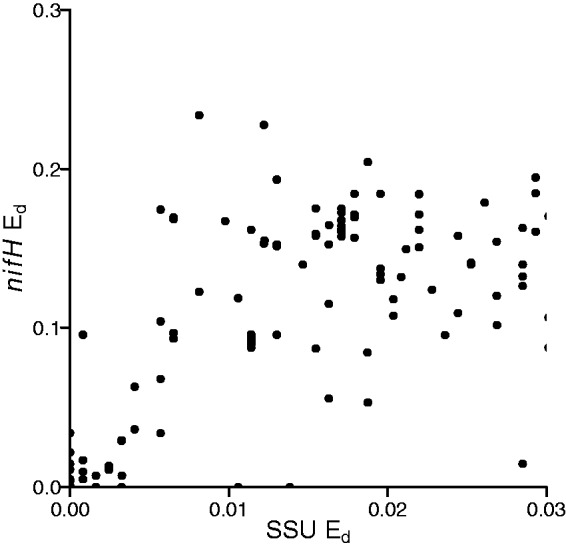
Comparison of E_d_ between 16S rRNA and *nifH* genes from sequenced genomes indicating levels of *nifH* divergence found among 16S rRNA gene sequences that have <3% sequence identity.

We also evaluated *nifH* sequence divergence among genomes that share >97% 16S rRNA sequence similarity (Figure 6). Microbial species are often delineated using a 97% 16S rRNA gene sequence similarity threshold (41). We observe that genomes that have >97% similarity in 16S rRNA genes can have up to 23% dissimilarity in their *nifH* sequences.

## Discussion

The multigene entries in the updated *nifH* database facilitate the evolutionary analysis of nitrogenase genes. We used the database to compare the E_d_ of *nifHDK* genes relative to each other and to 16S rRNA genes. This approach can demonstrate cases where horizontal gene transfer has occurred (40), evident as discontinuity on a plot of E_d_ values between a control and experimental gene. We observe partitioning between intra-domain and inter-domain (*Archaea* to *Bacteria*) comparisons of *nifH*, *nifD*, *nifK* relative to 16S rRNA genes (Figure 5). Such partitioning indicates horizontal gene transfer of the nitrogen-fixation genes between *Bacteria* and *Archaea*. This observation is consistent with previous evidence of such a transfer event (6, 16, 42).

We also compared E_d_ among *nif* genes to examine incongruence among the two principal *nif* clusters, cluster I and cluster III. There is partitioning of E_d_ values between *nif* genes from cluster I and cluster III (Figure 4). The E_d_ comparison for *nifD* and *nifK* (Figure 4C) shows a consistent accumulation of substitutions over evolutionary time. E_d_ comparisons that include *nifH* (Figure 4A and B) indicate discontinuity in the accumulation of mutations between cluster I and cluster III with respect to *nifH*. This discontinuity indicates that *nifH* has a distinct evolutionary history from *nifD* and *nifK*, having been horizontally transferred between clusters I and III at a point after *nifK* emerged from an ancient *nifD* (43).

Analysis of E_d_ values also indicated that the substitution rate in *nifD* is lower in cluster I than in cluster III (Figure 4A and C). In contrast, the substitution rate in *nifH* and *nifK* did not differ between clusters I and III. Differences in the substitution rate between gene sequence clusters indicate a difference in the evolutionary forces that shape protein evolution and are expected for proteins under different functional constraints. Models of nitrogenase protein structure indicate that the cluster III type nitrogenase interacts with a FeMo co-factor as would be expected if these genes are orthologous with cluster I type FeMo nitrogenase (*nif*) rather than either of the FeFe (*anf*) or FeV (*vnf*) alternative nitrogenases (44). In *nifD*, the active site where cofactor binding occurs displays sequence conservation according to the type of metal cofactor bound, with clusters I and III showing similar residues, although cluster III harbors an internal amino acid extension, which distinguishes it structurally from cluster I and the alternative nitrogenases (44). Another major distinguishing feature of cluster III versus cluster I is that cluster III contains strict anaerobes exclusively, whereas cluster I contains both aerobic organisms and facultative anaerobes. In addition, cluster III Fe–Mo nitrogenases are likely ancestral to both cluster I Fe–Mo nitrogenase and the alternative Fe–Fe and V–Fe nitrogenases (42). That mutations accumulate at different rates in the cluster III and cluster I type nitrogenases suggests that NifD is under different evolutionary constraints in these two groups. Further work is needed to determine the functional significance of NifD residues that differ between the cluster I and cluster III type nitrogenases and to identify functional constraints on nitrogenase evolution.

Our examination of E_d_ in *nifH* also has consequences for diversity analyses that use *nifH* as a marker gene. Sequence-based assessments of microbial diversity often use a similarity cutoff to delineate operational taxonomic units (OTUs) that are thought to correspond to species. A 97% identity cutoff is often applied to the 16S rRNA gene to define species level OTUs (45). In addition, genome sequence analysis has suggested that species level OTUs can be defined using a 95% identity cut-off for conserved protein encoding genes (46). However, ∼80% of nitrogen-fixing strains that share 97% 16S rRNA identity have <95% *nifH* identity and 43% have <85% identity (Figure 6). One pair of strains had >99% 16S rRNA identity but had *nifH* genes that differed in 23% of their nucleotide positions. Hence, measures of diversity based on *nifH* are not likely to be directly comparable with measures of diversity made with 16S rRNA genes. Furthermore, although it can be informative to discuss sequence diversity in *nifH*, it is not possible to use *nifH* sequence diversity as an estimate of species diversity.

There are two other collections of aligned *nifH* sequences that are publicly available. The Functional Gene Pipeline/Repository, known as FunGene, (33) provides an aligned collection of *nifH* sequences. The sequences in FunGene are pulled from public databases and aligned using HMMer. The FunGene sequence compilations are not in ARB database format, and these data are not incorporated into a phylogenetic context and lack sequence-associated metadata. A second *nifH* sequence collection is available from the Zehr research group (http://pmc.ucsc.edu/∼wwwzehr/research/database/) in ARB database format. This database is compiled using a set of representative NifH protein sequences using BLAST to identify NifH in public repositories. A major difference with the database we describe is the availability of linked *nifH*, *nifD*, *nifK* and 16S rRNA gene records from sequenced genomes. The database described herein also includes a comprehensive annotated phylogenetic tree of all *nifH* sequences and trees of the full-length sequences for *nifH*, *nifD*, *nifK* and 16S rRNA genes. These trees can be used to organize and navigate sequence data.

## Conclusions

The database we describe includes all *nifH* sequences present in the Genbank nucleotide database as of 16 May 2012 and includes associated metadata in a searchable framework. The *nifH* sequences are organized into phylogenetic trees that can be navigated readily in analyses of nitrogenase diversity and evolution and in the evaluation of *nifH* PCR primers. We demonstrate an application of the multigene functionality of the database to conduct evolutionary analysis of nitrogenase revealing evidence for horizontal gene transfer and substitution rate variation between the major phylogenetic clusters of nitrogenase. The database has utility for analysis of *nifH* genes generated as amplicons or shotgun fragments from metagenomic studies. The database is available in ARB format, a sequence database management tool used widely in microbial ecology, allowing for point-and-click access to sequence record importation and exportation, searching, alignment editing, primer design and matching and phylogenetic tree construction (35).

## Supplementary Data

Supplementary data are available at *Database* Online.
